# Estimation of the transmission of foot-and-mouth disease virus from infected sheep to cattle

**DOI:** 10.1186/1297-9716-45-58

**Published:** 2014-05-27

**Authors:** Carla Bravo de Rueda, Mart CM de Jong, Phaedra L Eblé, Aldo Dekker

**Affiliations:** 1 Central Veterinary Institute (CVI), Wageningen UR, P.O. Box 65, 8200 AB, Lelystad, The Netherlands; 2 Department Quantitative Veterinary Epidemiology, Wageningen University, P.O. Box 338, 6700 AH Wageningen, The Netherlands

## Abstract

The quantitative role of sheep in the transmission of foot-and-mouth disease virus (FMDV) is not well known. To estimate the role of sheep in the transmission of FMDV, a direct contact transmission experiment with 10 groups of animals each consisting of 2 infected lambs and 1 contact calf was performed. Secretions and excretions (oral swabs, blood, urine, faeces and probang samples) from all animals were tested for the presence of FMDV by virus isolation (VI) and/or RT-PCR. Serum was tested for the presence of antibodies against FMDV. To estimate FMDV transmission, the VI, RT-PCR and serology results were used. The partial reproduction ratio R_0_^p^ i.e. the average number of new infections caused by one infected sheep introduced into a population of susceptible cattle, was estimated using either data of the whole infection chain of the experimental epidemics (the transient state method) or the final sizes of the experimental epidemics (the final size method). Using the transient state method, R_0_^p^ was estimated as 1.0 (95% CI 0.2 - 6.0) using virus isolation results and 1.4 (95% CI 0.3 - 8.0) using RT-PCR results. Using the final size method, R_0_^p^ was estimated as 0.9 (95% CI 0.2 - 3.0). Finally, R_0_^p^ was compared to the R_0_’s obtained in previous transmission studies with sheep or cattle only. This comparison showed that the infectivity of sheep is lower than that of cattle and that sheep and cattle are similarly susceptible to FMD. These results indicate that in a mixed population of sheep and cattle, sheep play a more limited role in the transmission of FMDV than cattle.

## Introduction

Foot-and-mouth disease (FMD) is a contagious viral disease in cloven-hoofed animals caused by foot-and-mouth disease virus (FMDV). Clinical signs of FMD in sheep are frequently mild or not apparent [1]. But while sheep may not manifest clear clinical signs of FMD, they can secrete and excrete considerable amounts of FMDV [2-4] and therefore may play a significant role in FMDV transmission. Transmission of FMDV between sheep [5-8] and between cattle [9-11] has been studied previously. Transmission of FMDV from sheep to cattle may have occurred during the 1994 type O epidemic in Greece [12], during the 1999 type O epidemics in Morocco [13] and during the 2001 type O epidemics in UK [14]. However, transmission of FMDV from sheep to cattle has not yet been quantified.

In epidemiology, the reproduction ratio (R_0_) is an important quantitative parameter of transmission. R_0_ is defined as the average number of new infections caused by one typical infectious individual, during its entire infectious period, introduced into a population made up entirely of susceptible individuals [15]. Major outbreaks of FMDV can occur only if R_0_ is above 1. In the previously mentioned studies, R_0_ was estimated within species i.e. intraspecies transmission either in sheep or in cattle. When different species are mixed, the R_0_ for a mixed population of cattle and sheep not only depends on the occurrence of intraspecies (cattle-to-cattle and sheep-to-sheep) transmission but also on the occurrence of interspecies (sheep-to-cattle and cattle-to-sheep) transmission. To estimate R_0_ for a mixed population of cattle and sheep, all 4 (2 intraspecies and 2 interspecies) transmission parameters have to be known. The 2 interspecies transmission parameters will be called partial R_0_’s to emphasise that these parameters are strictly speaking not reproduction ratios. On the interspecies transmission of FMDV between sheep and cattle no quantitative information is available yet.

Moreover, with estimates for the intraspecies and interspecies (partial) R_0_’s, relative infectivity and susceptibility of sheep and cattle can be determined. Because for FMDV, relative infectivity and susceptibility have not extensively been quantified, modellers have had to rely on educated guesses about the relative infectivity and susceptibility of cattle, sheep and pigs herds [16]. Knowledge on relative infectivity and susceptibility of different species would improve modelling of FMDV transmission and more importantly could be used to implement tailored control measures in accordance to the animal species.

This study fills part of the gap on quantitative information on interspecies transmission of FMD. We estimated interspecies transmission of FMDV from infected sheep to contact cattle by estimating a partial R_0_ (R_0_^p^) for sheep to cattle transmission. Further, comparison of our results to those obtained in intraspecific transmission studies allowed us to define the relative infectivity and susceptibility of sheep and cattle.

## Materials and methods

### Experimental design

Twenty conventionally reared lambs (crossbred Texelaar-Noordhollander) aged between 6 and 7 months and 10 conventionally reared calves (pure- or crossbred (87%) Holstein-Frisian) aged between 6 and 8 months were used in this study. The study was performed in 10 separate animal rooms within the biosecurity facilities of the Central Veterinary Institute (CVI, Lelystad, The Netherlands). Each animal room was between 9 and 11 m^2^ in size. In each animal room, 2 infected lambs and 1 contact calf were housed together for 31 days. The study received ethical approval from the animal experiment committee of the CVI in accordance with Dutch law.

On the day of infection (0 days post infection (dpi)), all the lambs were moved to a separate animal room and inoculated with FMDV. Eight hours after inoculation, the lambs were reunited with their original roommates. The lambs were inoculated with FMDV strain Asia-1 TUR/11/2000 by intranasal instillation. The virus was obtained from the World Reference Laboratory for Foot-and-Mouth Disease (Pirbright, United Kingdom); it was passaged once in cattle before its use. The inoculum contained 10^5.8^ plaque forming units (pfu)/mL (tested on primary lamb kidney cells). Each lamb received 1.5 mL of inoculum per nostril.

### Sampling procedures

During animal inspection and/or sampling, animal caretakers changed coveralls and gloves between animal rooms. All the animals were inspected daily for clinical signs of FMD. In these inspections, rectal temperature above 39.5 °C in calves and above 40 °C in lambs was considered fever [17], and the animals were checked for the presence of vesicles and/or lameness. Oral swab samples were collected daily from each animal from 0 dpi until the end of the experiment (31 dpi). They were collected and processed as described previously [11], with the exception that we used medium containing 2% foetal bovine serum. The oral swab samples were stored at −70 °C until analysis by virus isolation (VI) and real time RT-PCR. Probang samples were collected from each animal at 29, 30 and 31 dpi. These were stored at -70 °C until analysis by real time RT-PCR. Heparinized blood samples were collected daily from each animal from 0 dpi until 11 dpi. The heparinized blood samples were centrifuged at 2500 RPM for 15 min; plasma was stored at −70 °C until analysis by VI. Samples for serum (clotted blood) were collected twice per week from 0 dpi till the end of the experiment (31 dpi). Serum was stored at −20 °C until serological analysis.

From the calves urine samples were collected daily during the first two weeks of the experiment and then twice per week until the end of the experiment. Urine samples were collected, as calves were stimulated to urinate spontaneously by rubbing the skin next to the vulva. In the laboratory, 800 μL of urine was mixed with 100 μL of foetal bovine serum and 100 μL of antibiotics (1000 U/mL of penicillin, 1 mg/mL of streptomycin, 20 μg/mL of amphotericin B, 500 μg/mL of polymixin B, and 10 mg/mL of kanamycin). Urine samples were stored at −70 °C until analysis by VI. From both animal species, faeces samples were collected from the rectum daily during the first two weeks of the experiment and then twice per week until the end of the experiment. Faeces samples were processed as described previously [18] with the exception that the samples were centrifuged at 3000 RPM for 15 min. The supernatants were stored at −70 °C until analysis by VI.

### Virus detection

All oral swab, heparinised blood, urine, and faeces samples were tested for the presence of FMDV as described previously [11], using plaque titration on monolayers of secondary lamb kidney cells (VI, i.e. detection of infectious virus particles). In addition all oral swab and probang samples were tested for the presence of FMDV using real time RT-PCR because in these samples neutralising antibodies, that could influence the virus isolation results, were expected to be present. RNA isolation was performed using the Magna Pure LC total Nucleid Acid Isolation kit (03 038 505) in the MagNa Pure 96 system (Roche®, Mannheim, Germany). Isolated RNA was tested as described previously [19] using a LightCycler 480 Real-Time PCR System (Roche®) with the exception that we used a Quantifast Probe RT-PCR Kit (Qiagen®, Venlo, The Netherlands).

### Serological analysis

The serum samples were tested for the presence of antibodies against both non-structural and structural proteins of FMDV. To detect antibodies against non-structural proteins, a PrioCHECK FMDV NS ELISA (Prionics®, Lelystad, The Netherlands) was performed in accordance to the manufacturers’ instructions. To detect antibodies against structural proteins, a virus neutralisation test (VNT) was performed as described previously [20], using the FMDV isolate Asia-1 TUR/11/2000 and Baby Hamster Kidney cells (BHK-21). Samples were considered to be positive when the VNT titres were above 10^0.6^ (VNT cut-off).

### Estimation of transmission parameters

#### Interspecies transmission rate

To estimate the transmission rate parameter β, which is the average number of new infections in a fully susceptible population caused by one typical infectious individual per unit of time [21], i.e. in our case the number of cattle (in a population of only cattle) that will become infected from one infectious lamb per day, we used a generalized linear model (GLM) [22]. The GLM was based on a stochastic SIR model [23] (in which infection dynamics are described by the change in number of susceptible (S), infectious (I), recovered (R) and total number (N) of animals). The GLM uses the number of new cases (of cattle in this case) as dependent variable and the total number of cattle as binomial total. The analysis is done with a complementary log-log (cloglog) link function, a binomial error term, and an offset as explained below [24].

The expression for the GLM is:

$$\mathrm{cloglog}\;E\left(C_{t}/S_{t}\right)=\ln\left(\beta \right)+\ln\left(I_{t}\Delta_{t}/N_{t}\right),$$

where ln(β) is the regression coefficient and ln(I_t_Δ_t_ /N_t_) is the offset variable.

E(C_t_/S_t_) = the expected number of cases (C_t_) during the interval (t,t + Δt) divided by the number of susceptible individuals (S_t_) at the start of the time interval (i.e. at t).

β = the transmission rate parameter.

I_t_ = the number of infectious animals at the start of time interval (t).

Δt = the duration of the time interval.

N_t_ = the total number of animals at the start of the time interval (t).

Note that because of the experimental design i.e. with all sheep infectious and all susceptible animals being cattle, the estimated β is an interspecies transmission rate parameter of sheep to cattle.

We assumed that the lambs were infectious from the first day until the last day FMDV was detected in their oral swab samples (by either VI or RT-PCR). Calves were considered infected if FMDV or antibodies against FMDV were detected in their samples. Because no virus was detected in 2 of the 4 contact calves that seroconverted, we assumed that both calves became infected 7 days before they scored positive in the VNT (which corresponded to the results from the calves that tested positive in VI and/or RT-PCR).

The data were analysed using the statistical program R [25]. The 95% confidence intervals (CI) of the estimated interspecies β were calculated using the standard error of the mean of log β.

#### Infectious period: T

We calculated the infectious period (T) based on the presence of virus in the oral swab samples from the individual lambs. Also for this purpose, both VI and RT-PCR results were used separately. The first moment at which an individual lamb tested positive in virus detection was considered as day 1 of its infectious period. The last day on which an individual lamb tested positive in virus detection (even if at one or more days in between no virus was detected), was considered as the last day of its infectious period.

Because some lambs still scored positive in virus detection at the end of the experiment, the mean duration of the infectious period T was calculated using a parametric (exponential) survival analysis [26]. To that end the time series of the lambs that scored positive in virus detection at the last day of the observational period were treated as censored data. The survival analysis was performed using the statistical program R [25] with the package “survival” [27]. The 95% confidence intervals (CI) of the estimated infectious period T were calculated using the standard error of the mean of log T.

#### Partial reproduction ratio: R_0_^p^

The partial reproduction ratio R_0_^p^ i.e. the average number of new infections caused by one infectious sheep, during its entire infectious period, when introduced into a population of susceptible cattle, was estimated using two different methods.

#### The transient state method

The transient state method takes the time course of the epidemic process into account [21]. We estimated the R_0_^p^ by multiplying interspecies β with the mean infectious period T, both estimated using VI and RT-PCR results. The 95% confidence intervals (CI) of the estimated reproduction ratio were calculated using exp (logβ + log T ± 1.96 · √ (var logβ + var log T) based on the assumption that the log transformed parameters follow a normal distribution and are independent.

#### The final size method

The final size method is based on the total number of infected calves at the end of the direct contact experiment, under the assumption that the epidemic process has ended before the experiment is stopped [21]. Even though some sheep (in contact to calves that did not become infected) were still shedding virus at the end of the experiment, we assumed that the epidemic process had ended at the end of the experiment. This assumption was based on the fact that FMDV transmission, leading to virus detection in the contact calves, occurred during the first week of the experiment (calf nr 5457 and calf nr 5463) at the moment when virus titres in oral swabs of sheep were high.

In a one-to-one experimental transmission design, the maximum likelihood estimate (MLE) of R_0_ (R_MLE_) can be derived analytically [21,28]. Because we used a two-to-one experimental transmission design, we derived the maximum likelihood estimate of $R_{0}:\;R_{\mathrm{MLE}}\;=\frac{3}{\sqrt{1-p}}-3$ where *p* is the total number of infection events divided by the number of independent replications. In the Additional files 1 and 2 the derivation of R_MLE_ is shown in more detail. The confidence intervals for *p* were derived from the binomial distribution. Consequently the confidence intervals for the final size R_0_^p^ could be calculated.

### Relative infectivities and susceptibilities of sheep and cattle

The relative infectivities and susceptibilities of sheep and cattle were determined by comparing the final size R_0_^p^ estimate obtained in this interspecies transmission study with the final size R_0_ estimates obtained in intraspecies transmission studies performed previously. The (intraspecies) final size R_0_ estimates used were: R_0__sheep-to-sheep_ = 1.1 [5,6] and R_0__cattle-to-cattle_ = ∞ [9], 2.52 [10], 14 (Bravo de Rueda et al., unpublished observations). By comparing R_0_^p^_sheep-to-cattle_ with R_0__sheep-to-sheep_, we could determine the relative susceptibility of sheep and cattle. By comparing R_0_^p^_sheep-to-cattle_ with R_0__cattle-to-cattle_, we could determine the relative infectivity of sheep and cattle.

## Results

### FMD clinical signs

In total 15 of the 20 inoculated lambs developed clinical signs of FMD (fever, vesicles and/or lameness). In lambs, fever (*n* = 13) was most frequently observed followed by vesicle formation (*n* = 11) and lameness (*n* = 10) (Table 1). Only one of the 10 contact calves (nr 5457) developed fever and had vesicles on the feet; the other 9 calves did not show clinical signs of FMD.

**Table 1:** Results of the virus isolation, RT-PCR, serology and clinical inspection.

	**Animal**		**Virus isolation**		**RT-PCR**		**Serology**		**FMD clinical signs**			**Contact infection**
**Room**	**Species**	**Nr**	**Oral swabs**	**Blood**	**Oral swabs**	**Probang**	**NS-ELISA**	**VNT**	**Fever**^**a**^	**Vesicles**	**Lameness**	
1	**Calf**	**5439**	-	-	-	-	-	-	-	-	-	No
	Lamb	5440	+	+	+	+	+	+	+	+	+	
	Lamb	5441	+	+	+	-	+	+	+	+	+	
2	**Calf**	**5442**	-	-	-	+	+	+	-	-	-	Yes
	Lamb	5443	+	+	+	-	+	+	+	-	-	
	Lamb	5444	+	-	+	+	+	+	-	-	-	
3	**Calf**	**5445**	-	-	-	-	-	-	-	-	-	No
	Lamb	5446	+	-	+	-	+	+	-	-	-	
	Lamb	5447	+	-	+	-	+	+	-	-	-	
4	**Calf**	**5448**	-	-	-	-	-	-	-	-	-	No
	Lamb	5449	+	+	+	+	+	+	+	+	-	
	Lamb	5450	+	+	+	-	+	+	+	-	-	
5	**Calf**	**5451**	-	-	-	-	-	-	-	-	-	No
	Lamb	5452	+	+	+	+	+	+	-	-	-	
	Lamb	5453	+	+	+	+	+	+	-	+	-	
6	**Calf**	**5454**	-	-	-	-	-	-	-	-	-	No
	Lamb	5455	+	+	+	-	+	+	+	+	+	
	Lamb	5456	+	+	+	+	+	+	+	+	+	
7	**Calf**	**5457**	+	+	+	-	+	+	+	+	-	Yes
	Lamb	5458	+	+	+	-	+	+	+	+	+	
	Lamb	5459	+	+	+	-	+	+	-	+	+	
8	**Calf**	**5460**	-	-	-	-	-	-	-	-	-	No
	Lamb	5461	+	+	+	-	+	+	+	-	+	
	Lamb	5462	+	+	+	-	+	+	+	-	+	
9	**Calf**	**5463**	-	-	+	-	+	+	-	-	-	Yes
	Lamb	5464	+	+	+	+	+	+	+	+	-	
	Lamb	5465	+	-	+	+	+	+	-	-	-	
10	**Calf**	**5466**	-	-	-	-	+	+	-	-	-	Yes
	Lamb	5467	+	+	+	-	+	+	+	+	+	
	Lamb	5468	+	+	+	+	+	+	+	+	+	

+/−, positive /negative in one or more of the tested samples.

^a^fever in sheep: body temperature above 40 °C; fever in cattle: body temperature above 39.5 °C.

### VI and RT-PCR

All the lambs tested positive for FMDV in oral swabs by VI. FMDV was first detected at 1–3 dpi. Higher levels of FMDV in oral swabs were detected in the first week after infection (Table 2). At the end of the experiment, oral swabs of 3 lambs (nr 5452, 5456 and 5458) still contained the virus. In total, 16 lambs tested positive by VI in their blood. Only 1 lamb (nr 5458) tested VI positive in its faecal sample. Only one calf (nr 5457) tested positive for FMDV in its oral swabs by VI (at 7–11 dpi). Virus was also isolated from blood and urine samples of this calf. No virus was isolated from faeces samples from any of the calves.

**Table 2:** FMDV virus titres in oral swab, blood, urine and faeces samples.

	**Days post infection**																																
**Animal**	**Nr**	**0**	**1**	**2**	**3**	**4**	**5**	**6**	**7**	**8**	**9**	**10**	**11**	**12**	**13**	**14**	**15**	**16**	**17**	**18**	**19**	**20**	**21**	**22**	**23**	**24**	**25**	**26**	**27**	**28**	**29**	**30**	**31**
**Calf**	**5439**	-^a^	-	tox^b^	-	-	-	-	-	-	-	-	N.A.^c^	-	-	-	-	-	-	-	-	-	-	-	-	-	-	-	-	-	-	-	-
Lamb	5440	-	2.6^d^	tox	3.8/V^d^	1.6	-	0.9	-	-	-	0.7	N.A.	-	-	-	1.1	-	-	1.7	1.1	0.7	-	1.0	-	-	1.2	-	1.4	-	-	-	-
Lamb	5441	-	1.3	tox	2.1/V	1.3	1.4	-	-	-	-	-	N.A.	-	-	-	-	-	-	-	-	-	-	-	-	-	-	-	-	-	-	-	-
**Calf**	**5442**	-	-	tox	-*^e^	-	-	-	-	-	-	-	N.A.	-	-	-	-	-	-	-	-	-	-	-	-	-	-	-	-	-	-	-	-
Lamb	5443	-	-	tox/V	2.8/V	- /V	1.0	0.4	-	-	-	-	N.A.	-	-	-	-	-	-	-	-	0.4	-	-	-	1.2	-	0.4	-	-	-	-	-
Lamb	5444	-	2.2	tox	-	0.4	1.0	0.9	-	-	-	1.0	N.A.	1.5	-	0.4	1.8	-	1.5	2.1	1.2	0.9	-	1.1	-	1.7	-	1.0	0.4	-	-	-	-
**Calf**	**5445**	-	-	tox	-	-	-	-	-	-	-	-	N.A.	-	-	-	-	-	-	-	-	-	-	-	-	-	-	-	-	-	-	-	-
Lamb	5446	-	0.4	tox	4.0	3.3	2.2	0.4	-	0.4	-	-	N.A.	-	-	1.0	1.1	-	2.5	N.A.	0.4	-	-	-	-	-	-	1.3	-	-	-	-	-
Lamb	5447	-	2.7	tox	0.4	2.4	2.4	-	-	-	-	-	N.A.	-	-	2.1	1.3	0.7	0.4	-	0.7	0.4	-	-	-	-	-	0.4	-	-	-	-	-
**Calf**	**5448**	-	-	tox	-	-	-	-	-	-	-	-	N.A.	-	-	-	-	-	-	-	-	-	-	-	-	-	-	-	-	-	-	-	-
Lamb	5449	-	2.1/V	tox/V	4.2/V	-	0.9	-	-	-	-	-	N.A.	-	-	-	-	-	-	-	-	-	-	-	-	-	-	-	-	-	-	-	-
Lamb	5450	-	3.7	tox/V	2.3/V	1.4	1.5	-	-	-	-	-	N.A.	-	-	-	1.7	-	1.2	0.4	-	1.7	-	-	-	2.1	-	1.0	-	-	-	-	-
**Calf**	**5451**	-	-	tox	-	-	-	-	-	-	-	-	N.A.	-	-	-	-	-	-	-	-	-	-	-	-	-	-	-	-	-	-	-	-
Lamb	5452	-	- /V	tox/V	4.0	1.6	-	0.4	-	-	-	-	N.A.	-	-	-	-	-	2.2	2.0	1.7	0.4	1.8	1.9	-	-	0.9	1.3	1.3	1.6	-	-	1.0
Lamb	5453	-	0.4/V	tox/V	2.3/V	1.9	1.4	1.1	-	-	-	0.7	N.A.	-	-	1.9	1.5	0.7	0.4	-	-	-	-	-	-	-	-	1.5	-	-	-	-	-
**Calf**	**5454**	-	-	tox	-	-	-	-	-	-	-	-	N.A.	-	-	-	-	-	-	-	-	-	-	-	-	-	-	-	-	-	-	-	-
Lamb	5455	-	-	tox	0.7/V	0.7/V	1.9	0.4	-	-	-	-	N.A.	-	-	1.4	0.9	1.4	1.5	1.4	-	-	-	0.9	-	-	-	-	1.4	-	-	1.2	-
Lamb	5456	-	1.6	tox	2.7/V	2.1/V	1.0	0.4	-	-	-	-	N.A.	-	-	1.0	0.4	-	0.9	1.4	1.5	-	-	0.4	-	-	-	0.9	-	-	1.7	-	0.7
**Calf**	**5457**	-	-	tox	-	-	-	-	3.1/V*	3.4/V	4.3/V^f^	4.3^f^	N.A.^f^	-	-	-	-	-	-	-	-	-	-	-	-	-	-	-	-	-	-	-	-
Lamb	5458	-	0.7	tox	3.9/V	0.9	0.9	1.2	-	-	- ^g^		N.A.	-	-	1.3	1.9	2.5	2.8	2.5	0.4	2.1	2.6	2.4	1.5	1.6	-	1.7	-	2.3	-	0.4	2.4
Lamb	5459	-	1.6	tox	2.8/V	2.2	0.4	-	-	-	-	2.3	N.A.	-	0.9	2.6	-	1.2	1.2	1.8	1.9	-	1.5	2.3	-	1.2	1.7	1.4	-	0.7	-	1.6	-
**Calf**	**5460**	-	-	tox	-	−	-	-	-	-	-	-	N.A.	-	-	-	-	-	-	-	-	-	-	-	-	-	-	-	-	-	-	-	-
Lamb	5461	-	0.4/V	tox/V	2.7/V	1.2	0.7	-	-	-	-	-	N.A.	-	-	0.4	1.4	-	0.9	-	-	-	1.2	1.7	-	-	-	-	-	-	-	-	-
Lamb	5462	-	2.1/V	tox/V	4.0/V	1.7	1.9	-	-	-	-	-	N.A.	-	-	-	-	-	-	0.9	-	-	-	1.1	-	-	-	-	-	1.6	-	-	-
**Calf**	**5463**	-	-	-	-	-	-	-	-	-	-	-*	N.A.	-	-	-	-	-	-	-	-	-	-	-	-	-	-	-	-	-	-	-	-
Lamb	5464	-	1.7	2.5	3.7	2.1/V	0.9/V	-	-	-	-	-	N.A.	-	1.7	1.5	1.2	2.6	0.9	1.2	2.3	-	-	1.8	-	1.2	2.2	2.5	1.7	0.4	-	-	-
Lamb	5465	-	-	2.1	-	0.4	2.2	-	-	-	1.5	-	N.A.	-	2.1	1.1	1.3	2.7	1.2	2.0	1.2	-	-	1.2	-	-	-	0.7	-	-	-	-	-
**Calf**	**5466**	-	-	-	-	-	-	-	-	-	-	-*	N.A.	-	-	-	-	-	-	-	-	-	-	-	-	-	-	-	-	-	-	-	-
Lamb	5467	-	- /V	3.0/V	2.7/V	2.8	1.8	-	-	-	-	-	N.A.	-	-	-	-	-	-	-	-	-	-	-	-	-	-	-	-	-	-	-	-
Lamb	5468	-	4.0/V	3.0/V	- /V	1.4/V	1.0	-	-	-	-	-	N.A.	-	-	1.0	0.9	0.4	1.2	0.4	-	-	1.8	2.0	2.1	-	-	-	-	-	-	-	-

^a^oral swab sample that scored positive for FMDV by virus isolation (VI) (log10 pfu/mL); −: no virus was detected.

^b^tox: toxic oral swab sample, no VI result available.

^c^N.A.: results not-available for oral swab samples.

^d^V = viraemia: blood sample that scored positive for FMDV by VI.

^e^* indicates the (estimated) day of infection of the contact calves 5442, 5457, 5463 and 5466.

^f^urine sample that scored positive for FMDV by VI.

^g^faeces sample that scored positive for FMDV by VI.

All the lambs tested positive for FMDV RNA in oral swabs by means of RT-PCR (Table 3). FMDV RNA in oral swabs was first detected at 1–2 dpi. At the end of the experiment, 8 lambs (nr 5446, 5447, 5452, 5455, 5456, 5458, 5461 and 5464) still tested positive for FMDV RNA in oral swabs. In total 9 lambs tested positive for FMDV RNA in their probang samples. Two of the 10 contact calves (nr 5457 and nr 5463) tested positive for FMDV RNA in oral swabs. Another contact calf (nr 5442) tested positive for FMDV RNA in one of its probang samples.

**Table 3:** FMDV RT-PCR results in oral swab samples.

	**Days post infection**																																
**Animal**	**Nr**	**0**	**1**	**2**	**3**	**4**	**5**	**6**	**7**	**8**	**9**	**10**	**11**	**12**	**13**	**14**	**15**	**16**	**17**	**18**	**19**	**20**	**21**	**22**	**23**	**24**	**25**	**26**	**27**	**28**	**29**	**30**	**31**
**Calf**	**5439**	-^a^	-	-	-	-	-	-	-	-	-	-	N.A.^b^										-	-	-	-	-	-	-	-	-	-	-
Lamb	5440	-	+	+	+	+	+	+	-	-	-	-	N.A.	-	+	+	+	+	+	+	+	+	+	+	+	+	+	+	+	-	-	+	-
Lamb	5441	-	+	-	+	+	+	+	-	-	-	-	N.A.	-	-	-	-	-	-	-	-	-	-	-	-	-	-	-	-	-	-	-	-
**Calf**	**5442**	-	-	-	-	-	-	-	-	-	-	-	N.A.	-	-	-	-	-	-	-	-	-	-	-	-	-	-	-	-	-	-	-	-
Lamb	5443	-	+	-	+	+	+	-	-	-	+	+	N.A.	-	+	-	+	-	-	-	-	+	-	-	+	+	-	+	-	-	-	+	-
Lamb	5444	-	+	+	-	+	+	+	-	-	+	+	N.A.	+	-	+	+	-	+	+	+	+	+	+	+	+	-	+	+	+	-	-	-
**Calf**	**5445**	-	-	-	-	-	-	-	-	-	-	-	N.A.	-	-	-	-	-	-	-	-	-	-	-	-	-	-	-	-	-	-	-	-
Lamb	5446	-	+	-	+	+	+	+	+	+	+	+	N.A.	+	-	+	+	+	+	N.A.	+	+	+	+	+	+	-	+	+	+	-	-	+
Lamb	5447	-	+	+	-	+	+	+	-	-	-	-	N.A.	-	-	+	+	+	+	+	+	+	+	-	+	-	-	-	-	-	-	-	+
**Calf**	**5448**	-	-	-	-	-	-	-	-	-	-	-	N.A.	-	-	-	-	-	-	-	-	-	-	-	-	-	-	-	-	-	-	-	-
Lamb	5449	-	+	+	+	+	+	+	-	-	-	-	N.A.	-	-	-	-	-	+	-	+	+	+	+	-	+	+	+	-	-	-	-	-
Lamb	5450	-	+	-	+	+	+	+	+	-	-	-	N.A.	+	-	+	+	-	+	+	-	+	+	+	-	+	+	+	-	-	-	-	-
**Calf**	**5451**	-	-	-	-	-	-	-	-	-	-	-	N.A.	-	-	-	-	-	-	-	-	-	-	-	-	-	-	-	-	-	-	-	-
Lamb	5452	-	-	+	+	+	-	+	-	-	-	-	N.A.	-	+	+	+	+	+	+	+	+	+	+	+	+	+	+	+	+	-	+	+
Lamb	5453	-	-	+	+	+	+	+	+	-	+	+	N.A.	+	+	+	+	+	+	+	-	+	-	+	+	+	+	+	+	+	-	+	-
**Calf**	**5454**	-	-	-	-	-	-	-	-	-	-	-	N.A.	-	-	-	-	-	-	-	-	-	-	-	-	-	-	-	-	-	-	-	-
Lamb	5455	-	+	+	+	+	+	-	-	-	-	-	N.A.	-	+	+	+	+	+	+	+	+	+	+	+	+	-	+	+	-	-	+	+
Lamb	5456	-	+	+	+	+	+	+	+	-	+	+	N.A.	+	+	+	+	+	+	+	+	-	+	+	+	+	+	+	-	+	+	-	+
**Calf**	**5457**	-	-	-	-	-	-	-	+	+	+	+	N.A.	+	+	-	-	-	-	-	-	-	-	-	-	-	-	-	-	-	-	-	-
Lamb	5458	-	+	-	+	+	+	+	-	-	-	+	N.A.	-	+	+	+	+	+	+	+	+	+	+	+	+	-	+	-	+	-	+	+
Lamb	5459	-	+	+	+	+	+	+	-	-	+	+	N.A.	+	+	+	+	+	+	+	+	+	+	+	+	+	+	+	-	+	-	+	-
**Calf**	**5460**	-	-	-	-	-	-	-	-	-	-	-	N.A.	-	-	-	-	-	-	-	-	-	-	-	-	-	-	-	-	-	-	-	-
Lamb	5461	-	+	-	+	+	+	-	-	-	-	-	N.A.	-	+	+	+	-	+	+	+	-	+	+	+	-	+	+	+	-	+	+	+
Lamb	5462	-	+	+	+	+	+	-	-	-	-	-	N.A.	-	-	+	-	+	+	+	-	-	-	-	-	+	-	-	-	+	-	-	-
**Calf**	**5463**	-	-	-	-	-	-	-	-	-	-	+	N.A.	+	-	-	-	-	-	-	-	-	-	-	-	-	-	-	-	-	-	-	-
Lamb	5464	-	+	+	+	+	+	-	-	-	+	+	N.A.	+	+	+	+	+	+	+	+	+	+	+	+	+	+	+	+	+	+	+	+
Lamb	5465	-	+	+	-	+	+	-	-	-	+	-	N.A.	+	+	+	+	+	+	+	+	+	-	+	+	+	-	-	-	-	-	+	-
**Calf**	**5466**	-	-	-	-	-	-	-	-	-	-	-	N.A.	-	-	-	-	-	-	-	-	-	-	-	-	-	-	-	-	-	-	-	-
Lamb	5467	-	+	+	+	+	+	-	-	-	-	-	N.A.	-	-	-	-	-	-	-	-	-	-	-	-	-	-	-	-	-	-	-	-
Lamb	5468	-	+	+	+	+	+	+	+	+	+	+	N.A.	+	+	+	+	+	+	+	+	+	+	+	+	+	+	+	-	+	+	+	-

^a^oral swab sample that scored positive for FMDV by RT-PCR; −: no virus was detected and +: virus detected.

^b^N.A.: results not-available for oral swab samples.

### Serological results

**Figure 1:**
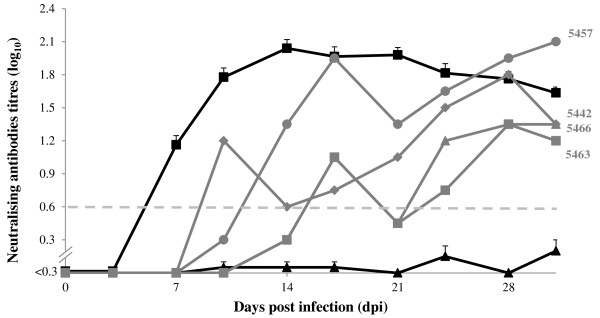
**Results of VNT titres.** The black line with solid boxes () represents the average VNT titres (log_10_) of the 20 inoculated sheep and the black line with solid triangles () the average VNT titres of the 6 VNT negative contact calves. For the 4 VNT positive contact calves (in grey lines) the individual VNT titres are shown. The grey dashed line () indicates the VNT cut-off (10^0.6^). Error bars represent the standard error of the mean.

Neutralising antibodies (by VNT) (Figure 1) were developed by all lambs, as were antibodies against non-structural proteins (by NS-ELISA) (Table 1). Neutralising antibodies were developed by four of the ten contact calves (Figure 1), these four calves also developed antibodies against non-structural proteins (Table 1) (calves nr 5442, 5457, 5463 and 5466). Calf 5457 became VNT positive at 14 dpi; 7 days after becoming positive in VI and RT-PCR. Calf 5463 became VNT positive at 17 dpi; 7 days after becoming positive in RT-PCR. Calf 5442 became VNT positive at 10 dpi and calf 5466 became VNT positive at 17 dpi. Figure 1 shows the averages of the VNT titres from the VNT positive lambs, the averages of the VNT titres from the VNT negative calves and the individual VNT titres from the 4 VNT positive contact calves.

### Estimation of transmission parameters

FMDV transmission occurred in 4 of the 10 groups. Calves 5457 and 5463 were detected infectious at 7 dpi and at 10 dpi respectively. Calves 5442 and 5466 did not test positive in any of the virus detection methods but they developed neutralizing antibodies at 10 and 17 dpi respectively. For the estimation of the transmission parameters, these calves were assumed becoming infected at 3 dpi and at 10 dpi respectively, 7 days prior to the detection of neutralising antibodies. The interspecies transmission rate parameter β, the infectious period T and the partial reproduction ratio R_0_^p^ were calculated using the results given in Tables 2 and 3.

Using the VI results, the interspecies β was estimated at 0.037 per day (95% CI: 0.014 - 0.076) and the infectious period T (of the sheep) was estimated at 28 days (95% CI 19. - 42.). Using the RT-PCR results, the interspecies β was estimated at 0.031 per day (95% CI: 0.012 - 0.065) and the infectious period T (of the sheep) was estimated at 46 days (95% CI 28. - 73.). By using the transient state method and the VI results, the R_0_^p^ was estimated to be 1.0 (95% CI: 0.20 - 6.0). By using the transient state method and the RT-PCR results, the R_0_^p^ was estimated to be 1.4 (95% CI: 0.30 - 8.0). By using the final size method, R_0_^p^ was estimated to be 0.90 (95% CI: 0.20 - 3.0). The estimated transmission parameters using the results from the VI and the RT-PCR analysis are shown in Table 4.

**Table 4:** Estimated transmission parameters using the results from the Virus isolation (VI) and the RT-PCR analysis.

	**Transmission rate parameter (interspecies β)**		**Infectious period (T)**		**Partial reproduction ratio (R**_**0**_^**p**^**)**			
	**β (day**^**−1**^**)**	**95% CI**	**T (days)**	**95% CI**	***Transient state method***		***Final size method***	
					**R**_**0**_^**p**^	**95% CI**	**R**_**0**_^**p**^	**95% CI**
**VI**	0.037	0.014 - 0.076	28.	19. - 42.	1.0	0.20 - 6.0		
**RT-PCR**	0.031	0.012 - 0.065	46.	28. - 73.	1.4	0.30 - 8.0		
							0.90	0.20 - 3.0

### Relative infectivities and susceptibilities of sheep and cattle

The estimated R_0_^p^_sheep-to-cattle_ is very similar to the R_0 sheep-to-sheep_ estimated previously (final size R_0_ = 1.1) in two intraspecies transmission studies with sheep [5,6], indicating that cattle and sheep are similarly susceptible to FMD.

The estimated R_0_^p^_sheep-to-cattle_ is lower than the R_0 cattle-to-cattle_ estimated previously in three intraspecific transmission studies with cattle (final size R_0_ = ∞ [9], final size R_0_ = 2.52 [10] and final size R_0_ = 14 in Bravo de Rueda et al., unpublished observation), indicating that cattle are more infectious than sheep.

## Discussion

The purpose of this study was to estimate transmission of FMDV from infected sheep to contact cattle and, together with results from previous studies, to identify differences in either susceptibility to FMD or infectivity of FMD infected sheep and cattle. Our study shows that FMDV transmission from sheep to cattle occurs, but the estimated partial reproduction ratio (R_0_^p^) indicates that the expected number of secondary cases in calves, caused by infected lambs, is relatively low. Moreover, the susceptibility of sheep to FMD seems to be similar to the susceptibility of cattle to FMD. This finding is supported by French et al. [29] who found overlapping distributions when analysing dose–response relationships in cattle and sheep exposed to FMDV in aerosols. The fact that cattle and sheep have a similar susceptibility to FMD and the fact that the transmission (R_0_) from cattle to cattle is higher than the transmission (R_0_^p^) from sheep to cattle, indicate that cattle are more infectious than sheep. Thus, cattle play the major role in the transmission of FMDV in a mixed population with sheep and cattle. These relative infectivities and susceptibilities are useful for modelling FMD spread such as for example in Backer et al. [16]. In their model they assumed that the susceptibility of cattle herds is twice the susceptibility of sheep herds. Our results can be used to update such FMD spread models, and more importantly, could be a reason to implement different control strategies for both animal species.

We estimated a partial reproduction ratio for sheep-to-cattle transmission. This estimate alone does not reflect transmission for an entire mixed population consisting of sheep and cattle. In such a population, cattle-to-cattle, sheep-to-sheep, sheep-to-cattle and cattle-to-sheep transmission can take place. For the estimation of transmission in a mixed population, more information and/or other mathematical techniques are required [15]. Even though sheep play a more limited role in transmitting FMDV as compared to cattle, the reproduction ratio in a mixed population of sheep and cattle can still be larger than 1, meaning that major outbreaks can occur. Probably, the R_0_ for a mixed population of cattle and sheep will be higher if a higher proportion of cattle are present.

Previously, we studied transmission of FMDV between cattle [9-11] and between sheep [6] using FMDV strain O/NET/2001. However, different strains of FMDV might affect different species and might have different transmission characteristics. In more recent studies, we therefore used another serotype of FMDV to study transmission of FMDV. We chose FMDV Asia-1 because this serotype spread towards mainland Europe [30,31]. We observed transmission of FMDV Asia-1 between sheep [5] and between cattle (Bravo de Rueda et al., unpublished observation), and now studied transmission between sheep and cattle. The R_0_ values obtained in the studies using serotype O and Asia-1 are not significantly different. Still, differences might exist for other serotypes.

In this study, we investigated within pen transmission. The animals in this study were in close proximity. Extrapolation of experimental data to field conditions should always be done with care. However, the relative infectivity and susceptibility will not change under field conditions. In field conditions, the estimated R_0_^p^ will probably be lower because it is known that between-pen transmission is lower than within-pen transmission [32-34]. Additionally, between-herd transmission will most likely be even lower [35].

The relative low R_0_^p^ in the transmission of FMDV from sheep to cattle can have implications for control measures implemented during an outbreak, e.g. whether or not to use vaccination in sheep, given the fact that vaccination against FMD is very effective in cattle [9,10]. If all cattle were vaccinated and thus became less infectious, then vaccination of sheep would not have an additional contribution to FMD control, especially when other control measures are implemented e.g. movement restrictions.

The observed relatively low infectivity of sheep is remarkable if we take into consideration that the duration of the secretion and excretion of FMDV in sheep (specifically in oral swabs) is much longer than in cattle. The mean duration of secretion and excretion of FMDV in sheep, in this study, was 28 days (VI results from oral swab samples). A similarly long period was shown by Eblé et al. [5], who showed that sheep secrete and excrete FMDV for longer than 30 days. In contrast, calves infected with the same strain of FMDV, secrete and excrete FMDV for on average 5.0 days (VI results from oral swab samples in Bravo de Rueda et al., unpublished observation). It was already known that sheep are long- term secretors and excretors of FMDV [3,36]. Nevertheless the results reported here show that this long-term secretion and excretion of FMDV in sheep does not enhance transmission of the infection from sheep to cattle. In our study, transmission events took place mainly during the first week after infection. This is in accordance with what others have observed in sheep [5-8] and in cattle [9,10].

In our study as well as in the above-mentioned studies, it was observed that FMDV is secreted and excreted in higher quantities during the first week post infection. Previous research showed that virus titres in upper respiratory tract samples from sheep are lower than those in cattle [4]. The ability of cattle to shed more virus than sheep could (partially) explain the observed difference in the infectivity of sheep and cattle. Moreover, in FMDV infected cattle, profuse salivation and nasal discharge are often observed [37]. Compared to cattle, salivation and nasal discharge after FMDV infection in sheep is less profuse i.e. sheep show less severe clinical signs [1,38,39]. Therefore it can be assumed that profuse secretion and excretion of the virus contributes to a higher contamination of the environment with FMDV. A recent analysis showed that FMDV transmission occurs for a large part through the environment (Bravo de Rueda et al., unpublished observations), and thus more new cases of FMD will take place if animals would shed more infected secretions and excretions.

We conclude that despite the ability of sheep to secrete and excrete FMDV for a relatively long period of time, sheep are less infectious than cattle. The observed differences in the relative susceptibility and infectivity of sheep and cattle could be a reason to implement different control strategies for both animal species.

## Competing interests

The authors declare that they have no competing interests.

## Authors’ contributions

CB participated in the design and coordination of the study, participated in the laboratory analysis, carried out the statistical analysis and drafted the manuscript. MJ participated in the design of the study, carried out the statistical analysis and drafted the manuscript. PE participated in the design and coordination of the study and helped to draft the manuscript. AD conceived the study, participated in the design and coordination of the study, carried out the statistical analysis and helped to draft the manuscript. All authors read and approved the final manuscript.

## Supplementary Material

Additional file 1 **Calculating reproduction ratio R**_**0 **_**in two-to-one transmission experiments.** This additional file shows the analytical derivation of the maximum likelihood estimate (MLE) of R_0_ (R_MLE_) in a two-to-one experimental transmission design. Click here for file

Additional file 2 **The two-to-one transmission experiment is graphically represented as an SI (susceptible-infected) plane.** This additional file is part of Additional file 1. This graph shows how a two-to-one transmission experiment can be represented using an infectious-susceptible plane. β is the transmission rate parameter, S_t_ is the number of susceptible animals, I_t_ is the number of infectious animals, N_t_ is the total number of animals and, α the recovery rate. Click here for file
