# Supplementary material for: Estimation of the transmission of foot-and-mouth disease virus from infected sheep to cattle
Source: Vet Res. 2014 May 27;45(1):58. doi: 10.1186/1297-9716-45-58 (PMC4058432; doi:10.1186/1297-9716-45-58)
Supplement: Additional file 1 — Calculating reproduction ratio R 0 in two-to-one transmission experiments. This additional file shows the analytical derivation of the maximum likelihood estimate (MLE) of R0 (RMLE) in a two-to-one experimental transmission design. [file 1297-9716-45-58-S1.docx]

**Additional file 1 Calculating reproduction ratio R_0_ in two-to-one transmission experiments**

**Introduction**

In disease transmission, quantifying the average number of newly infected individuals caused by an infectious individual during its entire infectious period (Reproduction ratio, R_0_) is important. When several infectious and susceptible individuals are mixed in an experiment, both infectious and susceptible individuals should have a similar status e.g. both be vaccinated, or e.g. be the same species. To avoid this problem transmission experiments can also be performed in a one-to-one transmission experiment set up [[21](#_ENREF_21),[24](#_ENREF_24)]. In one-to-one transmission experiments, the expression for R_0_ has been derived analytically, R_0_ = 2 p/(1-p) where *p* is the total number of infection events divided by the number of independent replications. Given the rates of infection and recovery (see Additional file 2), R_0_ is β times the infectious period (1/α) [[21](#_ENREF_21),[28](#_ENREF_28)]. Similarly to a one-to-one transmission experiment, in this paper we derive R_0_ for a two-to-one transmission experiment.

**Maximum Likelihood estimate for R_0_ in a transmission experiment using 2 infectious and 1 susceptible individuals**

The two-to-one transmission experiment can graphically be represented as an SI (susceptible-infected) plane. An additional file shows this graphical SI plane (see Additional file 2).

The probability that both infected individuals recover and the susceptible individual escapes infection (S_t_ = 1 and N_t_ = 3) is:

.

So the probability that the susceptible individual becomes infected is:

and .

Where
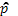
is the MLE of p, the total number of infection events divided by the number of independent replications and
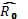
is the MLE of R_0_.

Note that in this case the estimated R_0_ is a partial reproduction ratio (R_0_^p^) as infectious animals are all sheep and susceptible animals are all cattle.
