# Supplementary figures and images for: Estimation of the transmission of foot-and-mouth disease virus from infected sheep to cattle
Source: Vet Res. 2014 May 27;45(1):58. doi: 10.1186/1297-9716-45-58 (PMC4058432; doi:10.1186/1297-9716-45-58)

## Slide 1
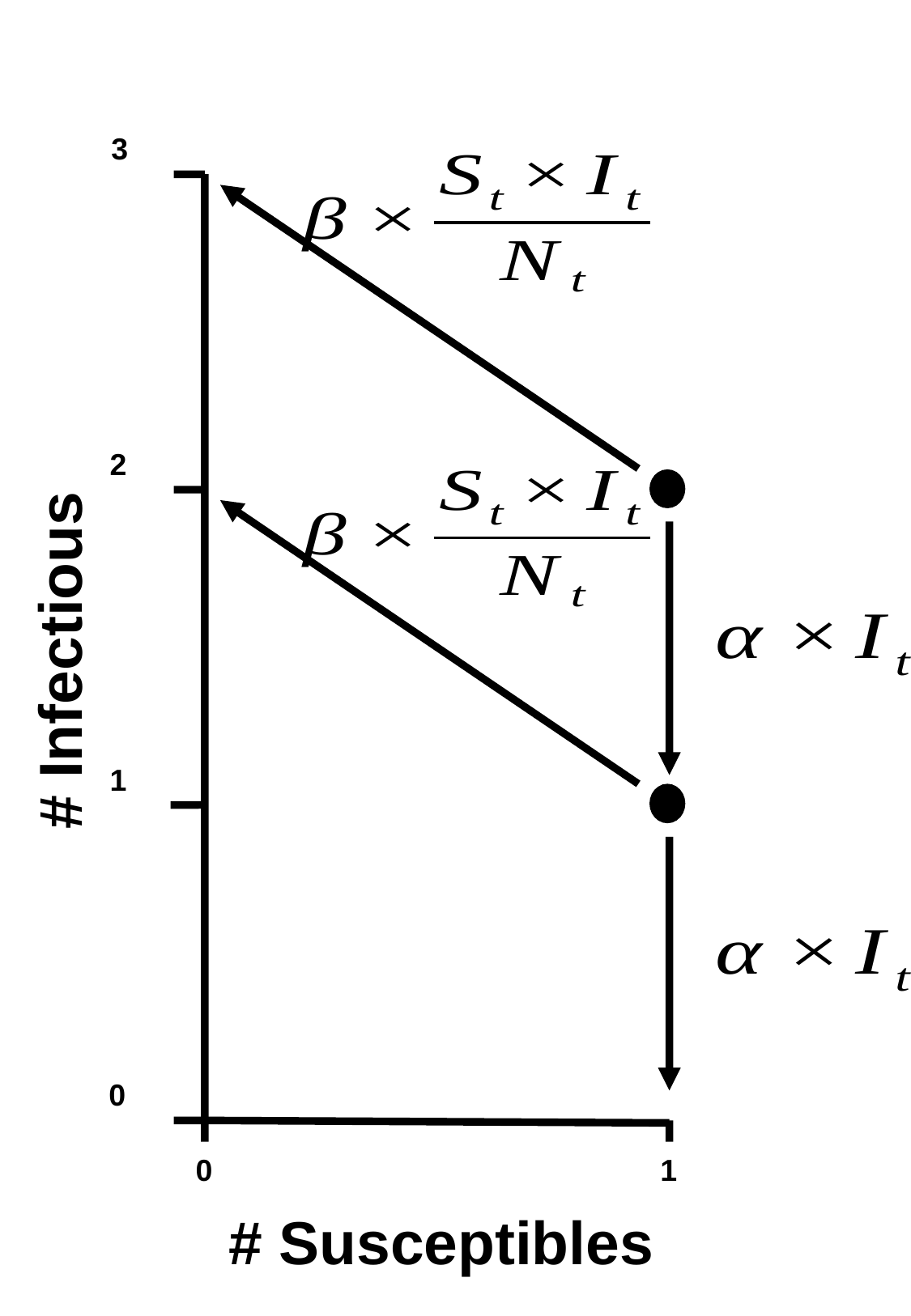

3
2
1
0
0
1
# Infectious
# Susceptibles

Supplement: Additional file 2 — The two-to-one transmission experiment is graphically represented as an SI (susceptible-infected) plane. This additional file is part of Additional file 1. This graph shows how a two-to-one transmission experiment can be represented using an infectious-susceptible plane. β is the transmission rate parameter, St is the number of susceptible animals, It is the number of infectious animals, Nt is the total number of animals and, α the recovery rate. [file 1297-9716-45-58-S2.ppt]
